# A Genetic Screen for Attenuated Growth Identifies Genes Crucial for Intraerythrocytic Development of *Plasmodium falciparum*

**DOI:** 10.1371/journal.pone.0013282

**Published:** 2010-10-11

**Authors:** Bharath Balu, Naresh Singh, Steven P. Maher, John H. Adams

**Affiliations:** Department of Global Health, College of Public Health, University of South Florida, Tampa, Florida, United States of America; BMSI-A*STAR, Singapore

## Abstract

A majority of the *Plasmodium falciparum* genome codes for genes with unknown functions, which presents a major challenge to understanding the parasite's biology. Large-scale functional analysis of the parasite genome is essential to pave the way for novel therapeutic intervention strategies against the disease and yet difficulties in genetic manipulation of this deadly human malaria parasite have been a major hindrance for functional analysis of its genome. Here, we used a forward functional genomic approach to study *P. falciparum* and identify genes important for optimal parasite development in the disease-causing, intraerythrocytic stages. We analyzed 123 *piggyBac* insertion mutants of *P. falciparum* for proliferation efficiency in the intraerythrocytic stages, *in vitro*. Almost 50% of the analyzed mutants showed significant reduction in proliferation efficiency, with 20% displaying severe defects. Functional categorization of genes in the severely attenuated mutants revealed significant enrichment for RNA binding proteins, suggesting the significance of post-transcriptional gene regulation in parasite development and emphasizing its importance as an antimalarial target. This study demonstrates the feasibility of much needed forward genetics approaches for *P. falciparum* to better characterize its genome and accelerate drug and vaccine development.

## Introduction

*Plasmodium falciparum* causes the most severe form of malaria and is responsible for much of the mortality associated with the disease [1]. A major obstacle to drug and vaccine development has been the inadequate comprehension of the higher value targets critical in the parasite's biology. Following genome sequencing of *P. falciparum*, several large-scale genomic studies have aided a great extent in understanding gene function [2], [3], [4], [5], [6], [7], [8], [9]. However, a considerable amount of the *P. falciparum* genome codes for genes that are conserved among, but unique to *Plasmodium* or other apicomplexan species. Presumably, such conserved hypothetical genes could provide most information about the parasite's unique biology and hence are arguably the best targets for antimalarial therapy.

The most direct way to study gene functions is to create gene knockouts through homologous recombination, followed by functional analyses. However, genetic manipulation of *P. falciparum* is an extremely challenging, tedious process due to a very low transfection efficiency (in the range of 10^−6^), the ability of the parasite to carry non-integrated plasmids as episomes and the inefficiency to recombine with the parasite genome [10]. It is therefore daunting to envision a whole-genome, homologous recombination-based knockout strategy for *P. falciparum*, as can be accomplished in model organisms, such as yeast [11].

In higher eukaryotes, random transposon mutagenesis has been used extensively to study gene functions through rapid generation of gene knockouts and knockdowns [12], [13], [14]. With the development of a highly efficient, *piggyBac* transposon-based mutagenesis system for *P. falciparum*, a large-scale, genome-wide insertion mutagenesis approach could aid greatly in dissecting the *P. falciparum* genome [15], [16].

Malaria parasite growth in the intraerythrocytic stages is determined by multiple factors: the length of the asexual cell cycle, the number of merozoites produced per schizont, the efficiency of merozoite release from host cells, and the invasion efficiency of new host cells by the newly formed merozoites. Each of these processes represents the net result of numerous molecular functions and may be possible targets for therapeutic intervention. In this study, we tested the feasibility of a forward genetic screen in *P. falciparum* by performing an *in vitro* proliferation assay for 123 *piggyBac*-transformed *P. falciparum* cloned lines to identify genes and pathways vital for intraerythrocytic development. This assay identified a high percentage of significantly attenuated growth mutants, which have insertions in genes of a variety of biological pathways, supporting a similar approach for large-scale whole genome screening.

## Results

**Figure 1:**
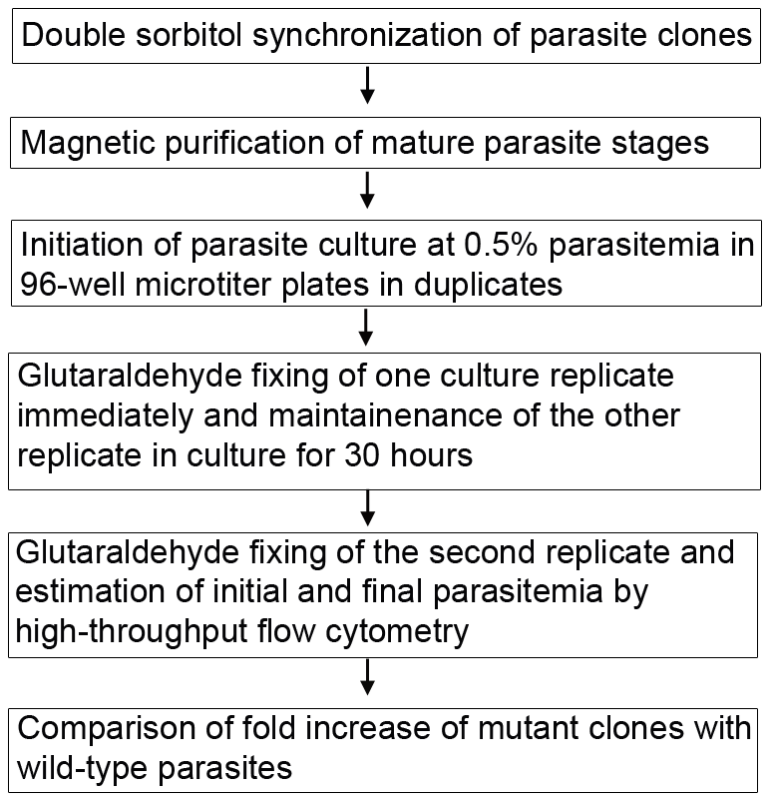
An *in vitro* proliferation assay for *Plasmodium falciparum*. A flowchart of steps involved in the *in vitro* proliferation assay for *Plasmodium falciparum* intraerythrocytic stages.

**Figure 2:**
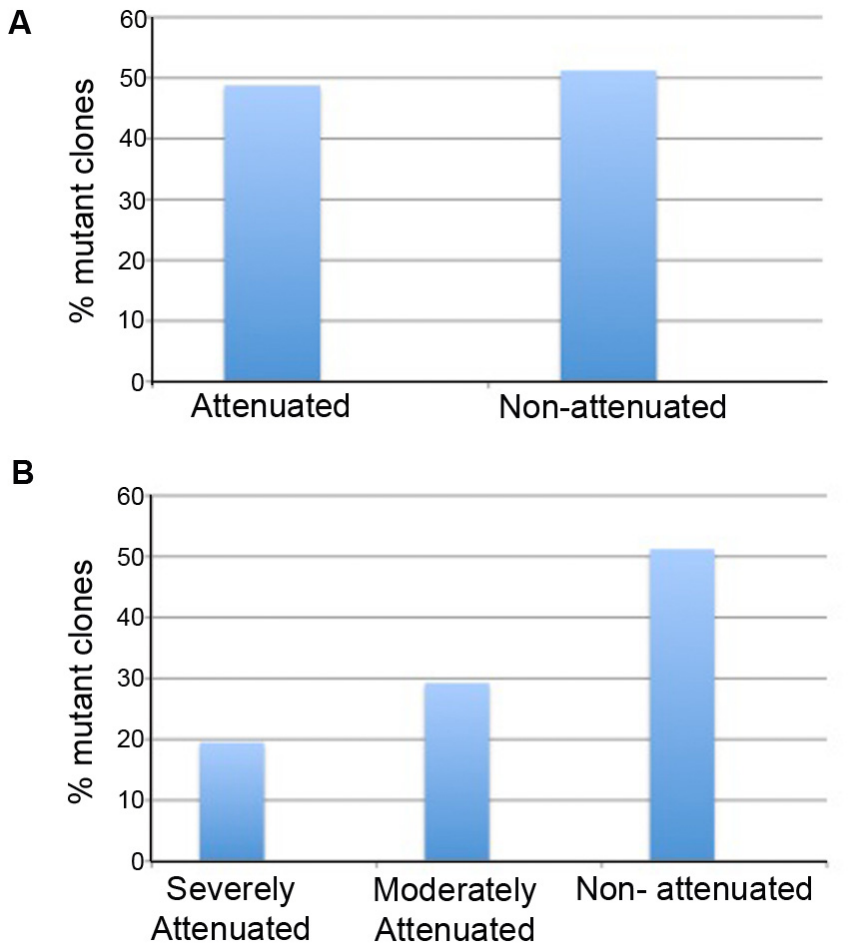
Attenuation of intraerythrocytic growth rate in mutant *Plasmodium falciparum* clones. (A) Categorization of *piggyBac*-transformed *P. falciparum* mutant clones as attenuated (with a reduced fold-change of >2 standard deviation (SD) from the mean of wild-type fold-change) and non-attenuated revealed attenuation of approximately 50% of the mutant clones. (B) Further sub-classification of the attenuated mutants as severely attenuated (with a reduced fold-change of >4 SD from the mean of WT fold-change) and moderately attenuated (with a reduced fold-change of 2>SD<4 from the mean of WT fold-change) showed severe attenuation of blood-stage growth in approximately 20% of the mutants.

To identify mutants that are attenuated or defective in intraerythrocytic growth, we used an *in vitro* proliferation assay to measure parasite fold-increase at the end of the asexual cycle, thereby estimating its efficiency to continue infection. In this assay, synchronized, mature, blood-stage parasites were allowed to invade fresh erythrocytes and parasitemia was measured before and after invasion by flow cytometry (Fig. 1). Parasite fold-increase was then calculated as a direct ratio between the parasitemia before and after invasion. Comparison of fold-change of mutant clones to wild-type (WT) parasites revealed that approximately 50% of the mutants (59 mutants) were significantly attenuated, with a reduced fold-change of >2 standard deviation (SD) from the mean of WT fold-change (*P*<0.05) (Fig. 2A) (Table S1). Based on the extent of attenuation, we further classified the mutants as severely attenuated (with a reduced fold-change of >4 SD from the mean of WT fold-change) and moderately attenuated (with a reduced fold-change of 2>SD<4 from the mean of WT fold-change) (Fig. 2B) (Table S1 and Table S2). Approximately 20% of the analyzed mutants (24 mutants) were severely attenuated and 30% (35 mutants) were moderately attenuated in intraerythrocytic proliferation (Fig. 2B). A few mutant clones displayed significantly higher proliferation rate than the wild-type parasites.

**Figure 3:**
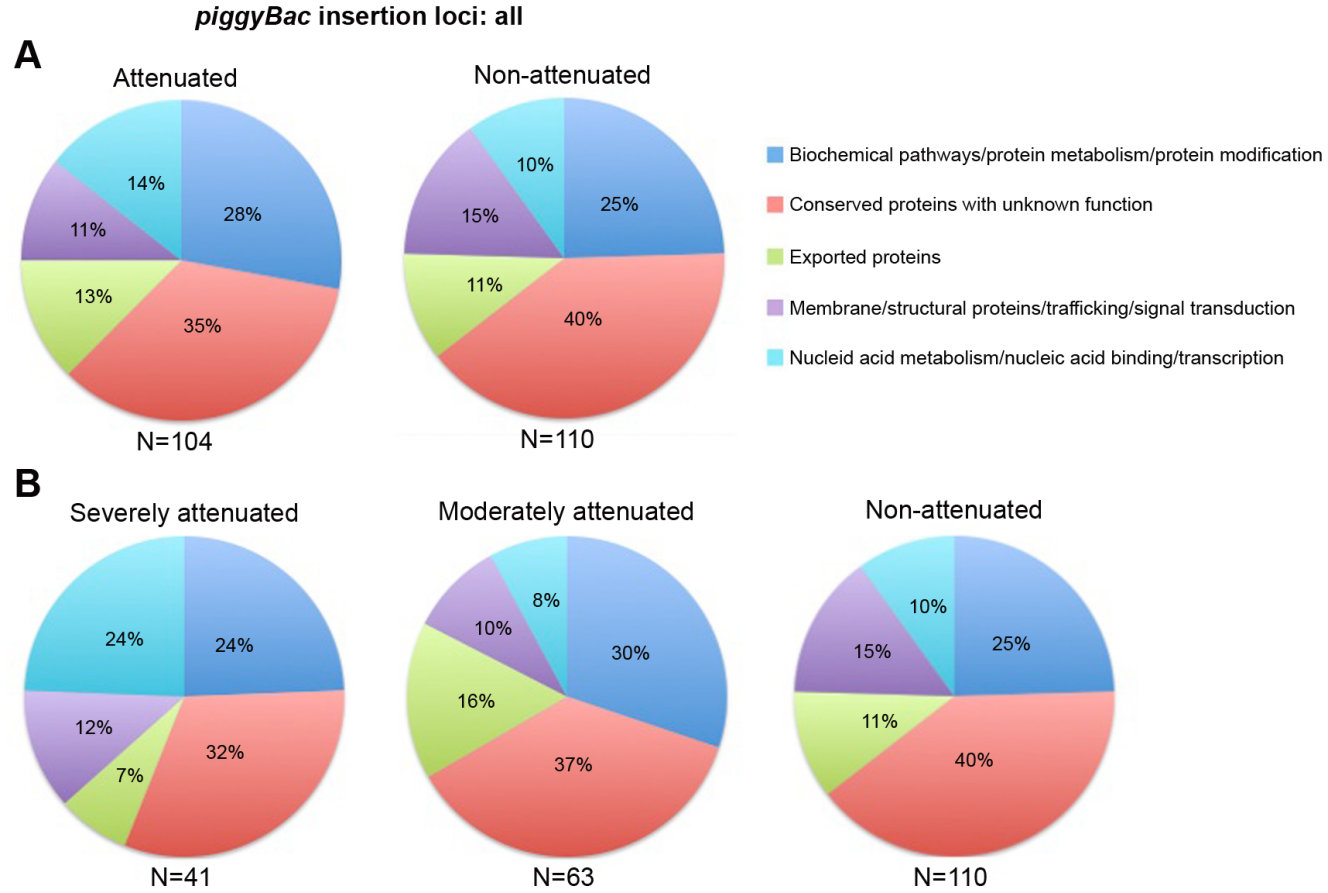
Functional categorization of genes immediately flanking *piggyBac* insertion sites. Functional distribution of all genes at *piggyBac* insertion loci did not show a difference between attenuated and non-attenuated mutants (A) however, following sub-classification of the attenuated mutants, genes involved in nucleic acid metabolism/nucleic acid binding/transcription were significantly enriched in the severely attenuated mutants (B).

To determine the existence of a bias for any functional category of genes in the attenuated mutants, all parasite genes were functionally classified into five broad categories based on their annotation in PlasmoDB [17] (Fig. 3A). First, we considered all genes immediately flanking the *piggyBac* insertion loci, regardless of their distance from the insertion site, and no significant differences were observed in functional categories affected in the attenuated mutants compared to the non-attenuated mutants (Fig. 3A). The analysis was repeated following sub-classification of attenuated mutants as severely and moderately attenuated, and genes involved in nucleic acid metabolism/nucleic acid binding/transcription were significantly enriched in the severely attenuated mutants compared to either moderately attenuated mutants (*P* = 0.02) or non-attenuated mutants (*P* = 0.04) (Fig. 3B) (Table 1).

**Table 1:** Functional categorization of all genes immediately flanking the *piggyBac* insertion loci shows significant enrichment for genes involved in nucleic acid metabolism/nucleic acid binding/transcription in the severely attenuated mutants (*P*<0.05).

Functional Category	Number of genes affected in each category		
	Severely attenuated	Moderately attenuated	Non-attenuated
Biochemical pathways/protein metabolism/protein modification	10	19	27
Conserved proteins with unknown function	13	23	44
Exported proteins	3	10	12
Membrane/structural proteins/trafficking/signal transduction	5	6	16
Nucleic acid metabolism/nucleic acid binding/transcription	10	5	11
**Total**	**41**	**63**	**110**

**Figure 4:**
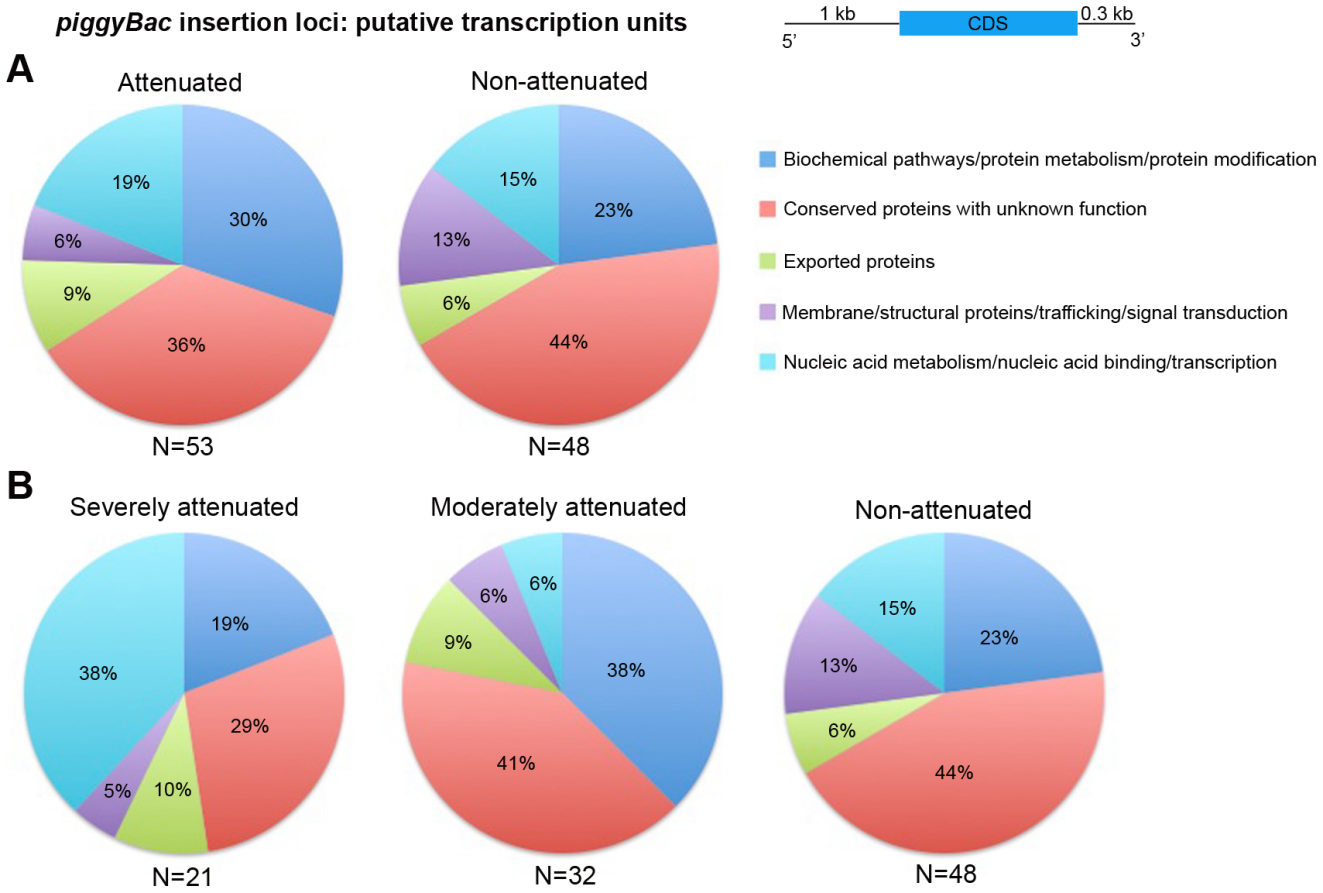
Functional categorization of transcriptional units immediately flanking *piggyBac* insertion sites. Functional distribution of transcriptional units (defined to include 1 kb sequence upstream to the coding sequence (CDS), the CDS and 0.3 kb downstream to the CDS) affected by *piggyBac* insertion did not reveal any differences between the attenuated and non-attenuated mutants as a whole (A) but showed significant enrichment of nucleic acid metabolism/nucleic acid binding/transcription genes in the severely attenuated mutants following sub-classification of the attenuated mutants (B).

Since close proximity of the *piggyBac* insertion to a gene's coding sequence is more likely to disrupt expression, we repeated the functional distribution analysis on genes, whose transcription units were most likely to be disrupted by *piggyBac* insertion. For this purpose, we defined the transcription unit of a gene to contain 1 kb sequence upstream to the ATG start codon and 0.3 kb sequence downstream to the stop codon (Fig. 4). As before, no significant differences were observed between the attenuated and non-attenuated mutants (Fig. 4A). However, sub-classification of the attenuated mutants again showed significant enrichment for genes in the nucleic acid metabolism/nucleic acid binding/transcription category in the severely attenuated mutants compared to the moderately attenuated mutants (*P* = 0.004) or the non-attenuated mutants (*P* = 0.03) (Fig. 4B) (Table 2).

**Table 2:** Functional categorization of putative transcriptional units affected by *piggyBac* insertion shows significant enrichment for genes involved in nucleic acid metabolism/nucleic acid binding/transcription in the severely attenuated mutants (*P*<0.05).

Functional Category	Number of genes affected in each category		
	Severely attenuated	Moderately attenuated	Non-attenuated
Biochemical pathways/protein metabolism/protein modification	4	12	11
Conserved proteins with unknown function	6	13	21
Exported proteins	2	3	3
Membrane/structural proteins/trafficking/signal transduction	1	2	6
Nucleic acid metabolism/nucleic acid binding/transcription	8	2	7
**Total**	**21**	**32**	**48**

The RNA binding proteins we identified as crucial, are predominantly involved in RNA processing and include a CAF1 family ribonuclease (MAL8P1.104), a DEAD-box helicase (MAL8P1.19), a forkhead-associated domain (FHA) containing RNA binding protein (PF11_0347), a pre-mRNA splicing protein (PFD0180c) and an RNA recognition motif (RRM) domain containing protein (PF13_0318) (Table 3). The most severe attenuation of any *piggyBac* mutant was caused by an insertion in the 5′region of PF13_0318, resulting in almost 75% reduction in proliferation rate versus wild-type NF54 parasites. In addition, three putative transcriptional regulators, two AP2 transcription factors and a histone acetyl transferase, were found to be extremely significant for intraerythrocytic growth (Table 3).

**Table 3:** List of genes involved in nucleic acid binding/nucleic acid metabolism/transcription identified to be crucial for intraerythrocytic development of *Plasmodium falciparum*.

Gene ID	PlasmoDB Annotation	Percent attenuation
PF13_0318	RNA binding protein	72
PFE0570w	RNA pseudouridylate synthase	57
PFF1100c	Transcription factor with AP2 domain(s)	56
PF13_0235	Transcription factor with AP2 domain(s)	50
PFD0180c	CGI-201 protein, short form	50
MAL8P1.104	CAF1 family ribonuclease	46
PFL1345c	RNA pseudouridylate synthase	46
PFL1350w	Histone acetyltransferase	46
PF11_0347	Conserved Plasmodium protein	43
MAL8P1.19	RNA helicase	41

## Discussion

A thorough understanding of the vital metabolic processes and pathways that enable malaria parasite's complex biology is significantly hindered due to the lack of functional characterization of its genome. Forward functional genomics approaches such as transposon-mediated insertion mutagenesis could provide valuable information about the parasite genome by associating a gene with its function, while perhaps more importantly deciphering essential from non-essential components of the genome. This study was aimed at testing the feasibility of such a screening approach in *Plasmodium falciparum* to identify genes crucial for intraerythrocytic growth of this parasite.

Using 123 genomic insertional mutants generated by random transposon mutagenesis [16], we performed a phenotype screen for reduced *in vitro* parasite proliferation rates in the parasite blood stages. Surprisingly, almost half of the mutants screened displayed significantly attenuated growth relative to the parent NF54 and 104 parasite genes were identified as potential regulators of intraerythrocytic development with 41 of them being crucial. In the case of mutants with *piggyBac* insertions in coding sequences, the attenuated growth phenotype could be directly attributed to the disrupted gene whereas in the case of insertions in between genes, further evaluation is required to identify the gene contributing to the attenuated phenotype. Functional classification of genes showed over-representation of genes involved in core processes regulating nucleic acids (nucleic acid metabolism/nucleic acid binding/transcription category) in the severely attenuated mutants, thus implying their significance in intraerythrocytic development and potential as high value targets for therapeutic intervention.

A majority of genes identified in this category of high value targets play a potential role in post-transcriptional gene regulatory mechanisms including five RNA binding proteins and two pseudouridylate synthases. The CAF1 family ribonuclease is a member of the eukaryotic CCR4-NOT complex, which is involved in global gene regulation and is the major cytoplasmic mRNA deadenylase in yeast [18], [19]. FHA domain is a phosphopeptide binding motif thought to function in nuclear signaling and is found in may protein kinases, transcription factors and RNA-binding proteins [20]. RRM domains can be found in heterogeneous nuclear ribonucleoproteins (hnRNPs), proteins regulating alternative splicing, protein components of small nuclear ribonucleoproteins (U1 and U2 snRNPs), and proteins that regulate RNA stability and translation [21]. Previous whole-genome analyses of *P. falciparum* recognized the relative abundance of RRM-containing proteins and the dominant role of RNA metabolism in the core processes of the interactome regulating gene expression [6], [22], [23]. Interestingly, two of the RNA binding proteins we have identified as vital for intraerythrocytic growth, PF13_0318 and PF08_0086, were previously identified as important interacting partners in an early computational model of the *P. falciparum* interactome [7]. PF08_0086 linked strongly with cyclophilins, implying its role in protein folding and trafficking. It also linked with PF13_0318, which in turn is linked to splicing factors and other RNA-binding proteins. Together, these two genes thus connect mRNA metabolism and protein synthesis to protein folding and trafficking [7]. Since transcriptome data shows PF13_0318 and PF08_0086 are expressed in all parasite stages at high levels, it seems reasonable to speculate that both genes play a significant housekeeping role in RNA metabolism, protein synthesis and protein trafficking throughout the life cycle [3], [4]. Our data provides the experimental support for the functional importance of these processes in the parasite and consequently the possible application of cyclosporin as an antimalarial drug [24].

Apicoplast and mitochondrial genes provide excellent targets for antimalarial therapy, as they are unique to the parasite. In our study we found that four apicoplast genes, PF07_0129, MAL8P1.86, PFD0710w and PFB0270w, and PFE1155c, a mitochondrial peptidase, cause considerable attenuation of parasite growth and provide grounds for further evaluation as antimalarial targets.

In conclusion, by using a forward genetics approach we have identified several genes that are extremely crucial for intraerythrocytic development of *Plasmodium falciparum* and provide an experimental basis for their further investigation as putative antimalarial targets. Furthermore, while this study focused on parasite growth rate in intraerythrocytic stages, other phenotypic screens for morphology, virulence, drug resistance, gametocytogenesis and transmission to mosquito hosts could be addressed with the mutant library, which will be available through the Malaria Research and Reference Reagent Resource Center (MR4) shortly.

## Materials and Methods

### Parasite Culture And Maintenance

*Plasmodium falciparum* wild-type NF54 clone and the mutant clones were cultured according to standard methods at 37°C and gassing (5% O2 and 5% CO2, nitrogen balanced) with 5% hematocrit (O+ blood, Interstate Blood Bank) in RPMI 1640 medium (Invitrogen) supplemented with and 0.5% Albumax II (Invitrogen), 0.25% sodium bicarbonate and 0.01 mg/ml gentamicin. Static cultures were maintained in all the experiments.

### Parasite Proliferation Assays

All parasite clones were synchronized twice at the ring stages 12 hrs apart using 5% sorbitol in a 5 ml culture. Following expansion to a 20 ml culture volume, mature parasite stages were purified on a magnetic column as described before [15]. Purified schizonts were counted using a hemocytometer and used to initiate new parasite cultures at 0.5% starting parasitemia. For each clone, the culture was plated into six wells of a 96-well microtiter plate, with 200 µl culture in each well. Immediately after plating, three of the six wells were fixed with 0.05% glutaraldehyde for flow cytometry and used to estimate the starting parasitemia in triplicates. Thirty hours later, the remaining three wells were fixed with 0.05% glutaraldehyde for flow cytometry and served as triplicates used to estimate the final parasitemia, post-invasion. The fold-change in parasitemia was then estimated as the ratio of final parasitemia to the starting parasitemia.

### Flow Cytometry

The flow cytometry protocol was adapted from previously described methods [25], [26]. In brief, each sample processed for flow cytometry contained 200 µl of parasite culture at 5% hematocrit in one well of a 96-well microtiter plate. Each parasite sample was fixed in 200 µl 0.05% glutaraldehyde in PBS after removal of culture medium, followed by permeabilization for 10 minutes in 0.3% triton-X 100, treatment for 1 hour with 0.5 mg/ml RNase A and staining for 1 hour with 0.1 mg/ml of ethidium bromide. The samples were then analyzed directly from the 96 well plate using a C6 Flow Cytometer® System ™ (Accuri cytometers) in a high throughput format. Following excitation with a 485 nm laser, forward size scattering (FSC) was used to count the total cell number and emission was recorded as peak area signals in FL-1 and FL-2 channels. The parasite-infected erythrocyte gates were defined by the population of cells with intense signals in the FL-2 channel that were distinct from uninfected erythrocytes. A total of 100,000 cells were counted for each sample. The data were analyzed using Cflow sampler software (Accuri cytometers).

### Statistical Analysis

In the proliferation assay, fold-changes in parasitemia were obtained in triplicates for wild-type and mutant parasites. The mean fold-change for each mutant was then tested for significant difference from the mean fold-change of wild-type parasites using a two-tailed student's *t* test. A *P* value of 0.05 was used as a cut-off for significant difference.

For comparison of functional categories of genes affected in attenuated and non-attenuated mutants, a Chi-Square test was performed (df = 1). A *P* value of 0.05 was used as a cut-off for significant difference.

## Supporting Information

Table S1 Functional classification of genes at insertion sites and relative distance of insertions to CDS.
(0.08 MB XLSX)

Table S2 Raw parasite growth data
(0.06 MB XLSX)
